# Interfacial Reinforcement Rather than Adsorption Quantity Determines the Stability of Soy Protein Isolate (SPI)/High-Methoxyl Pectin (HMP) Bilayer Emulsions

**DOI:** 10.3390/foods15183200

**Published:** 2026-09-10

**Authors:** Yi Liu, Jingyu Zhu, Jintao Wu, Ya Gao, Xiang Yu, Ying Kuang, Hong Qian, Kao Wu, Fatang Jiang

**Affiliations:** 1 Glyn O. Phillips Hydrocolloid Research Centre at HBUT, School of Life and Health Sciences, Hubei University of Technology, Wuhan 430068, China; liu.y@hbut.edu.cn (Y.L.); zhujingyu12355@163.com (J.Z.); wujintaozzw@163.com (J.W.); gaoya23333hh@163.com (Y.G.); yuxiang20010410@163.com (X.Y.); kuangying@hbut.edu.cn (Y.K.); jiangft@mail.hbut.edu.cn (F.J.); 2 School of Nursing and Health Management, Wuhan Donghu University, Wuhan 430212, China

**Keywords:** bilayer emulsions, QCM-D, emulsion stability, interfacial properties

## Abstract

Bilayer emulsions have gained increasing attention in modern food industry due to their tunable interfacial structure and resistance to environmental stresses. However, the mechanisms linking interfacial properties and macroscopic emulsion stability remain elusive. In this study, Soy Protein Isolate (SPI) and High-Methoxyl Pectin (HMP) bilayer emulsions with varied mass ratios were fabricated, and the relationship between interfacial properties and emulsion stability was systematically investigated. Laser diffraction particle size analysis, Confocal laser scanning microscopy, Turbiscan Stability and rheological characterization were explored to evaluate the emulsion stability, while quartz crystal microbalance with dissipation monitoring (QCM-D) was adopted to investigate interfacial adsorption behavior and the viscoelastic properties of the interfacial layers. The results demonstrated that the r = 1:2 formulation achieved the highest interfacial adsorption mass and greatest film thickness, whereas the r = 1:3 formulation exhibited the highest stability. The r = 1:3 ratio yielded the strongest binding affinity (Δ*f*_3_ = 15.02 Hz), energy dissipation (Δ*D*_3_ = 7.1941 × 10^−6^), and interfacial elasticity (43.15 kPa), indicating the formation of a highly hydrated and mechanically reinforced interfacial layer. The findings highlight that interfacial reinforcement efficiency rather than interfacial adsorption quantity is the key determinant governing the stability of SPI/HMP bilayer emulsions. This study would offer new insights into the structure–function relationship between interfacial characteristics and macroscopic stability in protein–polysaccharide bilayer emulsions, and provide theoretical guidance for the development of plant-based emulsion systems.

## 1. Introduction

Bilayer emulsions, with their tunable interfacial structures as well as facile and environmentally friendly fabrication process, have attracted increasing attention in the sustained release of active substances, lipid oxidation protection, and intelligent food delivery systems [1]. The mechanism of this technology mainly relies on electrostatic attraction. By sequentially depositing oppositely charged biopolymers onto pre-existing interface, layer-by-layer assembly enables the formation of a thick and dense interfacial films. Importantly, the composition and thickness of interfacial layers can be precisely controlled, enhancing resistance to environmental stress as well as functional delivery performance [2,3]. Compared to mixed emulsions, bilayer emulsions exhibits higher surface charges, thicker interfacial films, and denser interfacial structures, significantly improving the steric hindrance effect and electrostatic repulsion of the emulsion [4,5].

Among the various food-grade emulsifiers, protein–polysaccharide combinations are the most promising strategies due to their ability to provide electrostatic stabilization, steric hindrance, and excellent biocompatibility. SPI is an economical and commercially abundant plant protein with emulsifying capacity. Nevertheless, SPI is prone to molecular aggregation, and its limited interfacial flexibility often leads to flocculation and instability in the emulsion system [6,7]. High-Methoxyl Pectin (HMP), as a typical anionic polysaccharide, possesses long flexible molecular chains and high hydrophilicity. The HMP polymer chains can extend into the continuous aqueous phase to form a hydrated layer, providing strong steric stabilization and inhibiting interfacial protein aggregation [8,9]. Through electrostatic layer-by-layer assembly of protein and polysaccharides, a composite interfacial layer can be formed on the oil droplet surface, enhancing interfacial structure and improved stability [10]. However, this effect is strongly dependent on the SPI:HMP mass ratio: insufficient HMP may result in incomplete surface coverage or bridging between droplets, whereas excessive HMP may generate a diffuse, weakly retained interfacial layer or remain unadsorbed in the continuous phase. Consequently, optimization of the biopolymer ratio requires an understanding of how HMP loading modifies both interfacial assembly and emulsion-level stability [11,12].

Currently research on bilayer emulsions, has gradually shifted from improving the macroscopic stability of emulsions toward engineering interfacial microstructures [13,14,15]. Recent studies have demonstrated that tailoring interfacial structure through the co-assembly of proteins and polysaccharides could significantly improve emulsion stability [16,17]. However, these static descriptors alone cannot fully explain macroscopic emulsion behavior. Elucidating the relationship between layer-by-layer assembly and emulsion stability therefore requires techniques capable of resolving adsorption kinetics and structural evolution in real time [18,19]. Quartz Crystal Microbalance with Dissipation monitoring (QCM-D) is a highly sensitive interfacial characterization technique that enables real-time monitoring of adsorption mass and the evolution of viscoelastic properties at solid–liquid interfaces [20]. By simultaneously measuring changes in resonance frequency (Δ*f*) and energy dissipation (Δ*D*), QCM-D provides quantitative information on adsorption kinetics, interfacial mass loading, and the viscoelastic properties of adsorbed films [21]. Consequently, QCM-D has been widely employed to investigate the layer-by-layer assembly of biopolymers on model hydrophobic surfaces [22]. For instance, Li et al. examined the sequential adsorption of whey protein isolate (WPI) and low-methoxyl pectin (LMP) on hydrophobically modified gold sensors under different pH and ionic-strength conditions, whereas Wijaya et al. related the composition-dependent adsorption of heat-treated WPI–LMP complexes on a hydrophobic model surface to the properties of the resulting emulsions [20,21,22]. Nevertheless, it remains unclear how the protein-to-polysaccharide ratio governs sequential deposition, post-rinse layer retention, and macroscopic emulsion stability. This is essential for the formation of protein–polysaccharide bilayer emulsions.

In this study, SPI/HMP bilayer emulsions with 5% medium-chain triglyceride (MCT) as the oil phase were prepared via a two-step homogenization method based on electrostatic interaction. The microstructure, stability, and viscoelasticity of SPI/HMP bilayer emulsions with varied mass ratios were systematically investigated using confocal laser scanning microscopy (CLSM), stability analysis, laser particle size analysis, and rotational rheometry, whereas the layer-by-layer adsorption behavior of proteins and polysaccharides, and interfacial viscoelasticity changes were analyzed in real time using a quartz crystal microbalance (QCM-D). This study aimed to identify the interfacial characteristics that most consistently account for the rstability of SPI–HMP bilayer emulsions and to provide a mechanistic basis for their rational formulation.

## 2. Materials and Methods

### 2.1. Materials

Soy Protein Isolate (extra grade) from Shanghai Macklin Biochemical Technology Co., Ltd. (Shanghai, China). Citrus pectin (M_W_ = 2.21 × 10^5^ g/mol, galacturonic acid ≥74.0% (dried basis), ≤10% Moisture, degree of esterification (DE) value: 58.3%, P9135) was obtained from Sigma Co., Ltd. (Shanghai, China). Medium-chain triglycerides (analytical grade) were supplied by Yuanye Biotechnology Co., Ltd. (Shanghai, China). Nile blue dye (purity ≥75.0%) and Nile Red dye (purity ≥95.0%) were purchased from Macklin Co., Ltd. (Shanghai, China). Absolute ethanol (analytical grade) was purchased from Sinopharm Chemical Reagent Co., Ltd. (Shanghai, China). All chemical reagents are of analytical grade.

### 2.2. Preparation of SPI/HMP Bilayer Emulsion

The primary emulsion was prepared by adding 0.5 g of medium-chain triglycerides (MCT) to 4.75 g of 1% (*w*/*w*) Soy Protein Isolate (SPI) solution, followed by homogenization for 3 min at 19,000 rpm using an intermittent high shear homogenizer (F25, FLUKO Technology Development Co., Shanghai, China). High-speed shearing at 19,000 rpm was performed intermittently, using 1 min shearing periods separated by 30 s pauses to minimize heat accumulation. Under these conditions, the emulsion temperature did not exceed 45 °C throughout the homogenization process. Then 4.75 g of High-Methoxyl Pectin (HMP) solution (0%, 1%, 2%, 3% *w*/*w*) were added to the primary emulsion. The pH was adjusted to 3.9 using 1.0 mol/L hydrochloric acid, and the mixture was re-homogenized to form the SPI/HMP bilayer emulsion (final MCT concentration: 5% *w*/*w*). The SPI: HMP mass ratios in the final emulsion were 1:0, 1:1, 1:2, and 1:3.

### 2.3. Droplet Size, Size Distribution, and ζ-Potentials

The droplet size and size distribution were determined with a Mastersizer 2000 (Malvern Instruments, Malvern, UK). The refractive index of material was 1.47 and the absorption was set as 0.001. To determine ζ-potential, the emulsion samples were analyzed over an acidic-to-alkaline pH spectrum (2.0–8.0) employing a Malvern Zetasizer Nano ZS device (Malvern Instruments, Malvern, UK). A 4 mW He/Ne laser with an emission wavelength of 633 nm and a detection angle of 173° was used. Samples were diluted to 0.1 mg/mL with deionized water of equal pH and shaken to avoid multiple scattering effects.

### 2.4. Confocal Laser Scanning Microscopy (CLSM)

Confocal laser scanning microscopy (CLSM) (Leica Microsystems Inc., Heidelberg, Germany) was used to characterize the microstructure of the emulsions and the distribution of dispersion and continuous phases. According to a previous method [23], Nile Blue (1 mg/mL, dissolved in water) and Nile Red (1 mg/mL, dissolved in ethanol) were used as stains for the protein and oil components, respectively. The excitation wavelength of Nile Blue and Nile Red dyes were 633 nm and 488 nm, respectively.

### 2.5. Physical Stability

The physical instability behavior of the emulsions was assessed using a Turbiscan Lab Expert instrument (Formulaction, Toulouse, France). A 25 mL volume of the fresh SPI:HMP bilayer emulsion was poured into a cylindrical glass tube and monitored continuously over a period of 6 h at 6-min intervals. Changes in backscattering intensity (ΔBS) along the vertical height of the sample and the Turbiscan Stability Index (TSI) curve over time were recorded to characterize the emulsion’s instability.

### 2.6. Rheology Behaviors for SPI/HMP Bilayer Emulsion

The rheological properties of the SPI/HMP bilayer emulsion were evaluated using a dynamic rheometer (Mars 60, Thermo Fisher Scientific, Germany) equipped with 60 mm parallel plates, following a previously established method with slight modifications [24].

The temperature was maintained at 25 ± 1 °C, and the gap between the plates was fixed at 0.05 mm. Apparent viscosity was measured over a shear rate range of 0.1 to 100 s^−1^ when 1 mL of the emulsion was loaded on the measuring plate. Based on the amplitude sweep results, a strain of 1.0% was selected for subsequent testing. A time sweep test (γ = 1.0%, *f* = 1 Hz, duration of 1800 s) was performed to evaluate the short-term time-dependent rheological behavior of the emulsion.

### 2.7. QCM-D Measurement

Based on the methodology of previous studies [25], the adsorption behavior of SPI/HMP complexes using a quartz crystal microbalance with dissipation monitoring (QCM-D; model Q-Sense E1, Biolin Scientific, Västra Frölunda, Sweden). The surface hydrophobicity of gold sensors (QSX 301, 4.95 MHz, Biolin Scientific, Västra Frölunda, Sweden) was evaluated using a contact angle goniometer, and sensors exhibiting a water contact angle of approximately 104 ± 2.0° were selected for the experiments. After a stable baseline was established with buffer, a 0.1% (*w*/*w*) SPI solution was introduced onto the hydrophobic sensor until the frequency and dissipation responses reached a stable plateau (stage I). The sensor was subsequently rinsed with buffer to remove bulk-phase and loosely associated SPI (stage II). HMP solutions at concentrations of 0.1, 0.2, and 0.3% (*w*/*w*) were then introduced to obtain the nominal SPI formulations of r = 1:1, 1:2, and 1:3, respectively (stage III). For the SPI-only control (r = 1:0), buffer without HMP was introduced during this stage. Finally, the sensor was rinsed again with buffer to evaluate the retention of the adsorbed interfacial layer (stage IV). The r refers to the nominal ratio based on the respective feed-solution concentrations rather than the actual interfacial mass ratio adsorbed on the sensor. After establishing a stable baseline by rinsing the sensor with ultrapure water, the sample solution was injected until the frequency reached a plateau. Subsequently, the sensor was rinsed with ultrapure water to remove unabsorbed or weakly bound emulsions. The flow rate was set at 50 μL/min throughout the experiment, with shifts in frequency (Δ*f*) and energy dissipation (Δ*D*) being continuously recorded. Information regarding the viscoelastic properties of the adsorbed layer formed on the sensor surface was derived via the Voigt model, through which the layer thickness and mass were calculated.

The normalized frequency (Δ*f*_n_/n) and dissipation (Δ*D*_n_) responses at the 3rd, 5th, 7th, 9th, 11th, and 13th overtones were analyzed. Data were acquired using QSoft 401 and fitted using QSense Dfind. The bulk-fluid density and viscosity were fixed at 1000 kg m^−3^ and 1.0 × 10^−3^ Pa·s, respectively, while the density of the hydrated adsorbed layer was fixed at 1200 kg m^−3^ [20]. In general, rigid adsorbed layers are amenable to analysis using the Sauerbrey model, whereas viscoelastic films must be analyzed using the Voigt model [26,27]. To obtain more quantitative information, we fitted the raw data at multiple overtone numbers to the Voigt model according to the method described by Reviakine et al. [27].

### 2.8. Statistical Analysis

All experimental procedures were performed in triplicate. One-way analysis of variance (ANOVA) was used to evaluate differences among mean values, followed by Tukey’s multiple-comparison test at a significance level of *p* < 0.05. Data visualization conducted on Origin 2018 (Origin lab Corporation, Northampton, MA, USA) as well as SPSS 25.0 (IBM, Chicago, IL, USA).

## 3. Results and Discussion

### 3.1. The Droplet Size and Zeta-Potential of Bilayer Emulsions

**Figure 1:**
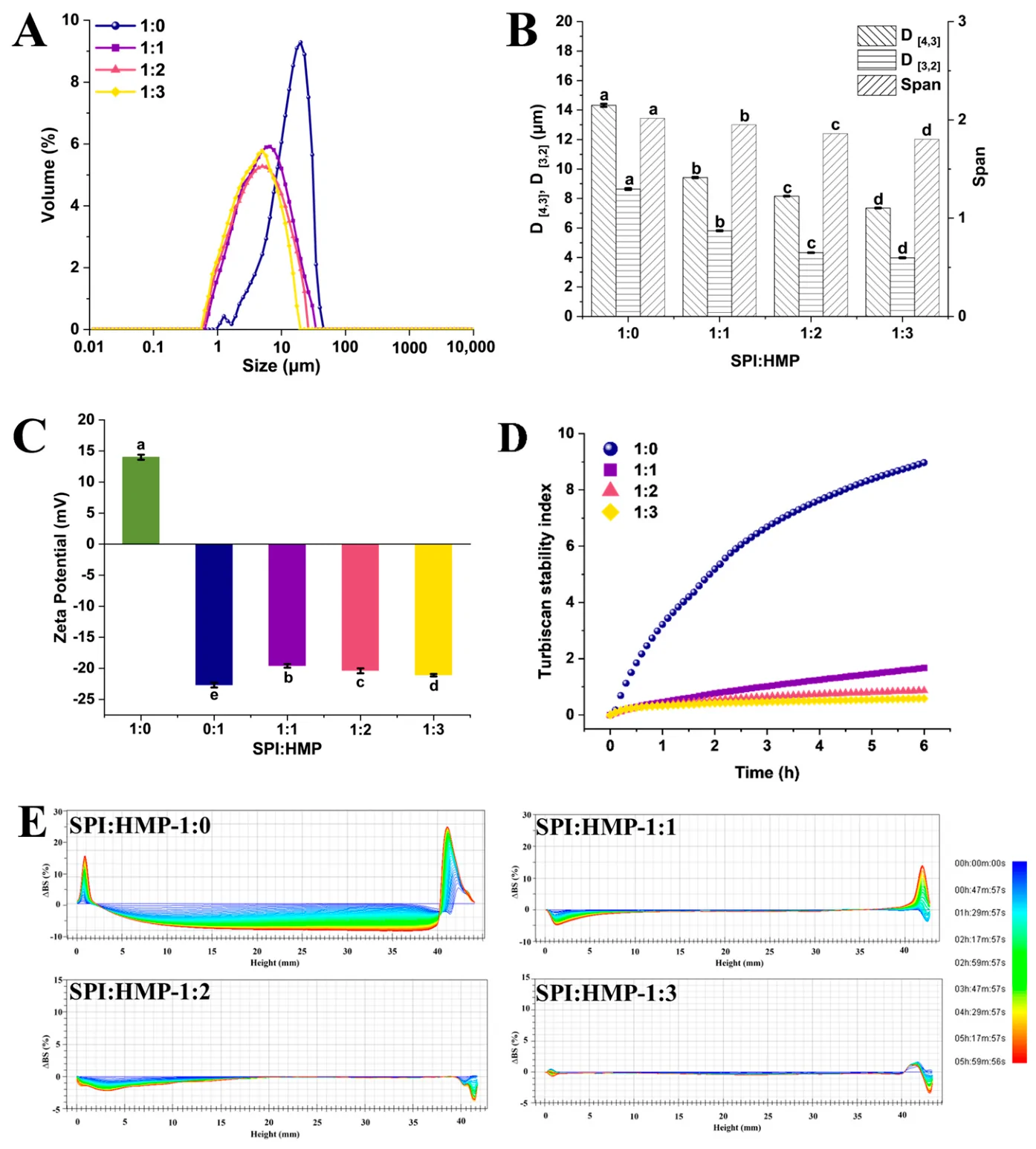
Droplet size distribution (**A**), Span (**B**), zeta potential (**C**), TSI value (**D**) and △BS (**E**) of SPI/HMP bilayer emulsions with varied ratios. Different letters indicated significant differences (Different lowercase letters indicate significant difference, *p* < 0.05).

The droplet size distributions of SPI/HMP bilayer emulsions with varied mass ratios were presented in Figure 1A,B, and the corresponding quantitative parameters including D _[4,3]_, D _[3,2]_, Span and zeta potential are summarized in Table 1. The emulsion stabilized by SPI only displayed a bimodal peak size distribution, indicating pronounced droplet aggregation and heterogeneous dispersion. However, the SPI/HMP emulsion exhibited a uniform and narrow unimodal peak, suggesting improved droplet uniformity. It was noted that with incorporation of HMP, the droplet size of the SPI/HMP bilayer emulsion decreased significantly, with the smallest diameter (D _[3,2]_ = 3.974 ± 0.027 μm) at r = 1:3. These results demonstrated that HMP effectively enhances the interfacial stabilization capability of SPI-stabilized emulsions due to the formation of an SPI/HMP bilayer interface through electrostatic interactions. The isoelectric point of SPI is approximately 4.2–4.5. At pH 3.9, SPI molecules hadn’t sufficient electrostatic repulsion to droplet aggregation [28]. HMP, as an anionic polysaccharide-based emulsifier, adsorbed onto the SPI-coated droplet surface through electrostatic attraction, forming a secondary layer [29]. This increased the thickness and hydrophilicity of the interfacial layer, providing enhanced steric hindrance and electrostatic repulsion between droplets. Moreover, hydrated HMP chains may establish a spatial network surrounding the droplets, restricting droplet collision and coalescence, contributing to improved long-term physical stability and a more homogeneous droplet distribution [30].

Zeta potential, defined as the potential difference between the slipping plane and the bulk aqueous phase surrounding dispersed droplets, is widely used to evaluate the electrostatic stability of emulsion systems [31]. The electrostatic repulsion between droplets can effectively inhibit flocculation and coalescence, thereby improving dispersion stability [32]. As shown in Figure 1C, the zeta potential of SPI/HMP bilayer emulsions varied significantly with increasing HMP concentration. The absolute value of zeta potential increased gradually (*p* < 0.05), reaching the maximum value of −21.1 ± 0.2 mV at an SPI:HMP ratio of 1:3. At pH 3.9, SPI exhibited a positive charge due to the pH being below its pI, whereas HMP carried abundant negatively charged groups. The increase in HMP concentration promoted the formation of a more complete polysaccharide-rich interfacial layer, leading to charge reversal and enhanced negative surface potential. This reduced the probability of droplet aggregation, enhancing emulsion stability. The trend was consistent with the result of droplet size, confirming that HMP helped interfacial reinforcement of stabilizing SPI/HMP bilayer emulsions [33].

### 3.2. Microstructure of Bilayer Emulsions

**Figure 2:**
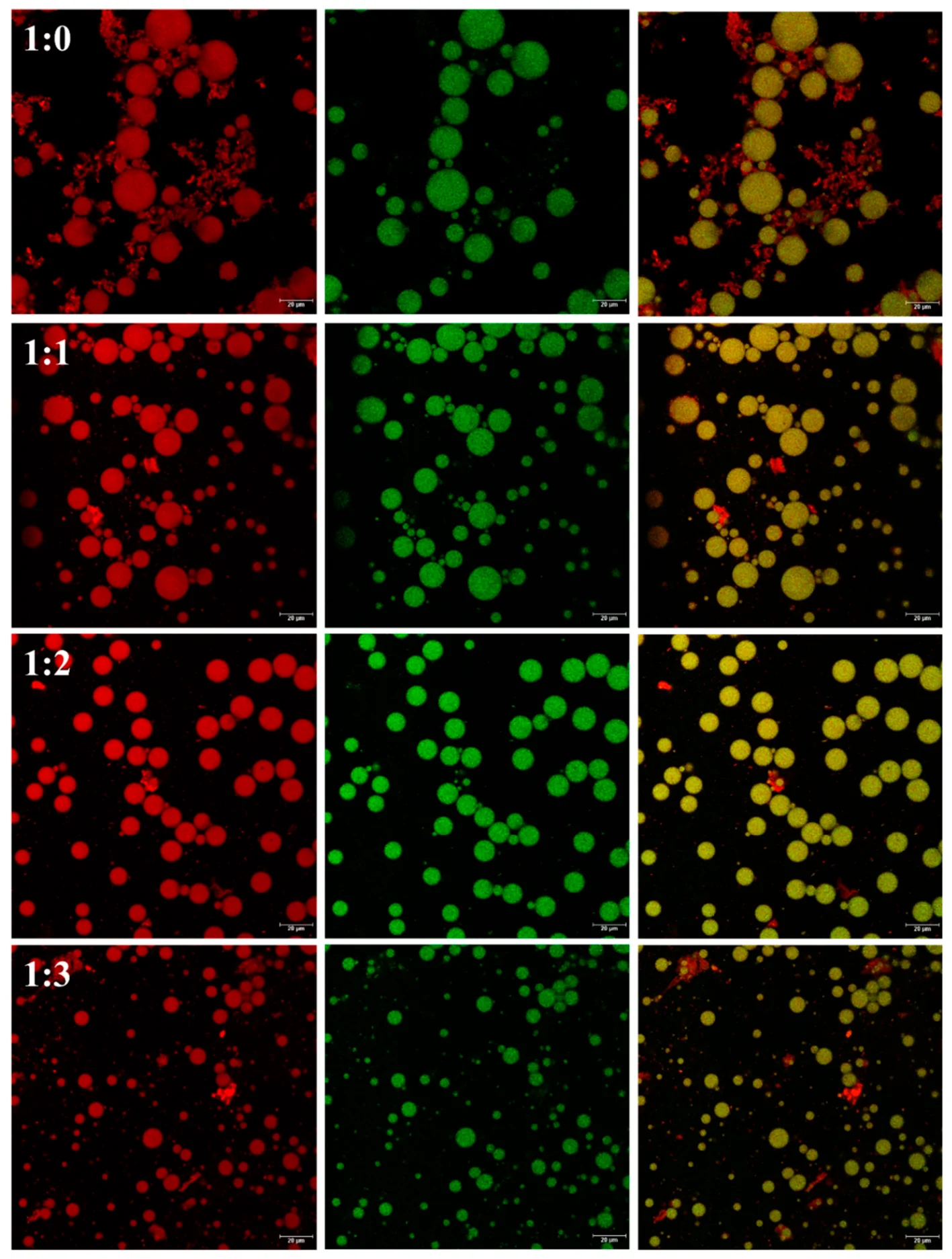
Confocal laser scanning microscopy (CLSM) images of SPI/HMP bilayer emulsions with varied ratios. The protein phase was stained with Nile Blue (red), and the oil phase was stained with Nile Red (green); the merged overlay images are shown in yellow (scale bar = 20 μm).

Confocal laser scanning microscopy (CLSM) was employed to investigate the microstructure of the emulsions and visualize the spatial distribution of oil droplets and interfacial proteins [34]. Figure 2 shows the CLSM images of SPI emulsions and SPI/HMP bilayer emulsions prepared at different SPI: HMP mass ratios, where green fluorescence represents oil droplets and red fluorescence corresponds to proteins. In the SPI-stabilized emulsion, relatively large oil droplets with an uneven distribution and evident flocculation were observed, indicating insufficient interfacial stabilization and a high tendency toward droplet aggregation. After the incorporation of HMP, the oil droplets became markedly smaller and more homogeneously dispersed, accompanied by enhanced protein/polysaccharide interfacial coverage. This enhancement can be attributed to the electrostatic attraction between positively charged SPI and negatively charged HMP under acidic conditions, which facilitates the formation of an SPI/HMP interfacial bilayer. Additionally, the hydrated HMP chains may provide additional steric stabilization, contributing to suppressing flocculation [35]. It was noted that the emulsion (r = 1:3) exhibited the smallest and most uniformly droplet distributed. This indicated that higher HMP concentration offered more complete interfacial coverage and steric protection. The hydrated network as well as the compact interfacial layer formed by HMP restricted droplet aggregation and enhanced dispersion stability. The results were in agreement with the droplet size results (Figure 1B).

### 3.3. Analysis of Bilayer Emulsion Stability Process

The destabilization process of the bilayer emulsion was characterized with a Tur-biscan measurement [36,37]. As depicted in Figure 1E, the relative backscattered light intensity (ΔBS) across the sample column height evolves over time for SPI/HMP bilayer emulsions. Compared with the SPI-stabilized emulsion, the SPI/HMP bilayer emulsions exhibited smaller fluctuations in backscattering intensity. It was attributed that HMP suppressed droplet movement and enhanced resistance to phase separation. Moreover, the emulsion (r = 1:3) showed the lowest variation in ΔBS, indicating the strongest ability to resistance against destabilization [38]. It can be attributed to the formation of an HMP-reinforced interfacial layer through electrostatic attraction between SPI and HMP.

The Turbiscan Stability Index (TSI) was adopted to quantitatively assess emulsion stability [39]. As shown in Figure 1D, The SPI-only emulsion exhibited rapid destabilization, with the TSI value increasing to 8.97 within 6 h. After the incorporation of HMP, the TSI values decreased markedly, reaching the lowest value (0.58) at r = 1:3. The result was consistent with the droplet size results, further confirming that HMP effectively improved the stability of the bilayer emulsion due to the enhanced interfacial protection. The high physical stability at r = 1:3 was a synergistic outcome of interfacial electrosteric repulsion and continuous-phase kinetic arrest.

### 3.4. Rheological Properties of Bilayer Emulsions

**Figure 3:**
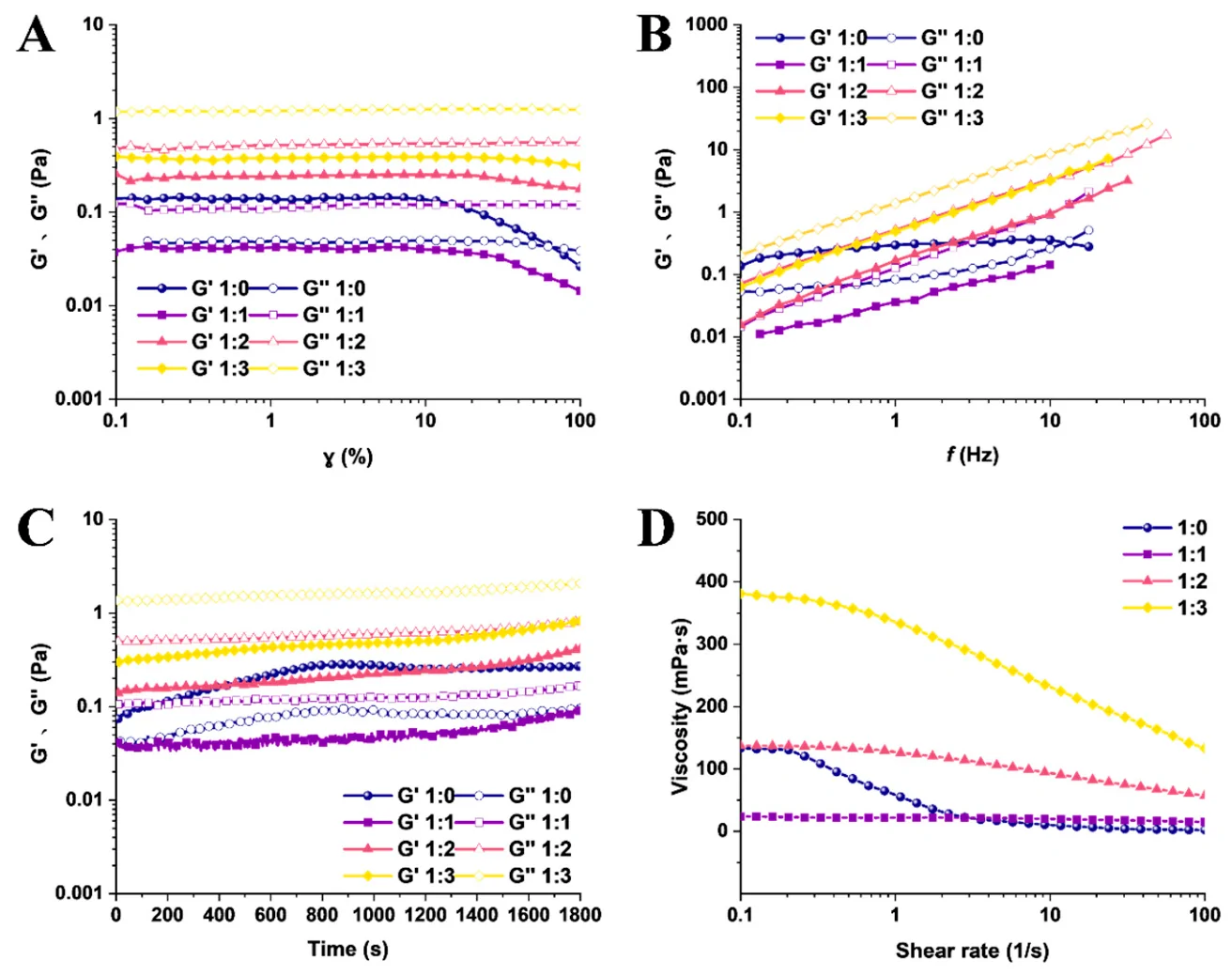
Rheological behavior (**A**–**C**) and Viscosity (**D**) of SPI/HMP bilayer emulsions with varied ratios.

The rheological behavior can not only clarify the interaction mechanism of various components in emulsion system, but also reveal the relationship between interfacial molecular structure and material mechanics [40]. The effect of HMP concentration on the dynamic strain scans of SPI/HMP bilayer emulsions is shown in Figure 3A. At a lower strain, both storage modulus (G′) and loss modulus (G″) of the SPI/HMP bilayer emulsions remained constant, exhibiting a highly linear viscoelastic behavior as well as relative intact microstructure. As the strain cross the critical amplitude, both G′ and G″ dropped considerably. The SPI/HMP bilayer emulsion exhibited a highly nonlinear viscoelastic behavior. The linear viscoelastic region (LVR) of the SPI emulsion was the smallest, *γ* = 7.2% strain. Correspondingly, with the addition of HMP, the LVR range of the SPI/HMP bilayer emulsion expanded, reaching a maximum of 30.4%. It demonstrated that HMP effectively improved the deformation resistance of the emulsion. This correlated with the formation of the interfacial layer between HMP and SPI via electrostatic interaction, enhancing the stability under external stress. It was noted that the crossover of G′ and G″ was beyond the LVR, suggesting structural reordering or flow of emulsion droplets induced by the applied stress and an increase in the dominating viscous behavior [29].

The G′ and G″ versus shear frequency was summarized in Figure 3B. For SPI emulsions, G′ was higher than G″ at low frequencies, whereas G″ was higher than G′ at high frequencies. It indicated that that SPI emulsions formed a weak elastic network at low frequencies, However, it enhanced viscous dissipation under rapid deformation at high frequencies. In contrast, higher values of G′ and G″ were observed after the incorporation of HMP, implying the bilayer interface enhanced the viscoelasticity of the emulsion. It may be attributed that HMP hydration formed the dense networks, limiting the mobility of the emulsion droplets and improving the viscosity and modulus values [41]. Additionally, the emulsion (r = 1:3) exhibited the highest G′ value. This demonstrated that a higher concentration contributed to the more rigidity interfacial structure.

As shown in Figure 3C, continuous oscillations of about 1800 s were applied to the emulsions at a strain amplitude (*γ* = 1.0%) and a fixed frequency (*f* = 1 Hz) to evaluate the short-term time-dependence of different ratios of SPI/HMP bilayer emulsions. For the SPI emulsion, a large increase in both G′ and G″ was observed within 600 s, indicating its strong short-term time dependence and relatively poor resistance to rearrangement. However, the G′ and G″ values of the SPI/HMP emulsions remained relatively stable, because of the HMP that improved structural stability. Moreover, the loss tangent (tan δ = G″/G′) for all SPI/HMP bilyer emulsions remained above 1 but decreases with time. This demonstrated that the emulsions were viscous-dominated system. As the interfacial network stabilized, the elasticity increases with time, contributing to the long-term emulsion stabilization.

Steady-state flow behavior provides insight into emulsion stability. As described by the Stokes equation, increased viscosity directly reduces the droplet settling velocity (v), thereby improving kinetic stability of the emulsion [42]. All emulsions showed a decrease in viscosity as the shear rate increased from 0.1 to 100 s^−1^) (Figure 3D), demonstrating pseudoplastic characteristics [43]. This was associated with the disruption of weak inter-droplet interactions, the deformation of droplet networks, as well as the alignment of polymer chains under shear. The apparent viscosity decreased in the order: r = 1:3 > r = 1:2 > r = 1:1 > r = 1:0. It was mainly attributable to the two factors: (i) the high hydration ability of HMP increased the viscosity of the continuous phase of emulsions; (ii) the increased HMP content promoted stronger electrostatic interactions with SPI, promoting the formation of the thicker interfacial layer that further enhanced the resistance to coalescence.

### 3.5. QCM-D Analysis

QCM-D was used to monitor the sequential adsorption of SPI and HMP on a planar hydrophobically modified gold surface using a 0.1% (*w*/*w*) SPI feed solution. This surface served as a model hydrophobic substrate. A sequential adsorption strategy was applied to construct the SPI/HMP bilayer interface, consisting of four consecutive stages: (I) initial SPI adsorption, (II) first rinsing, (III) secondary HMP adsorption, and (IV) second rinsing [44].

**Figure 4:**
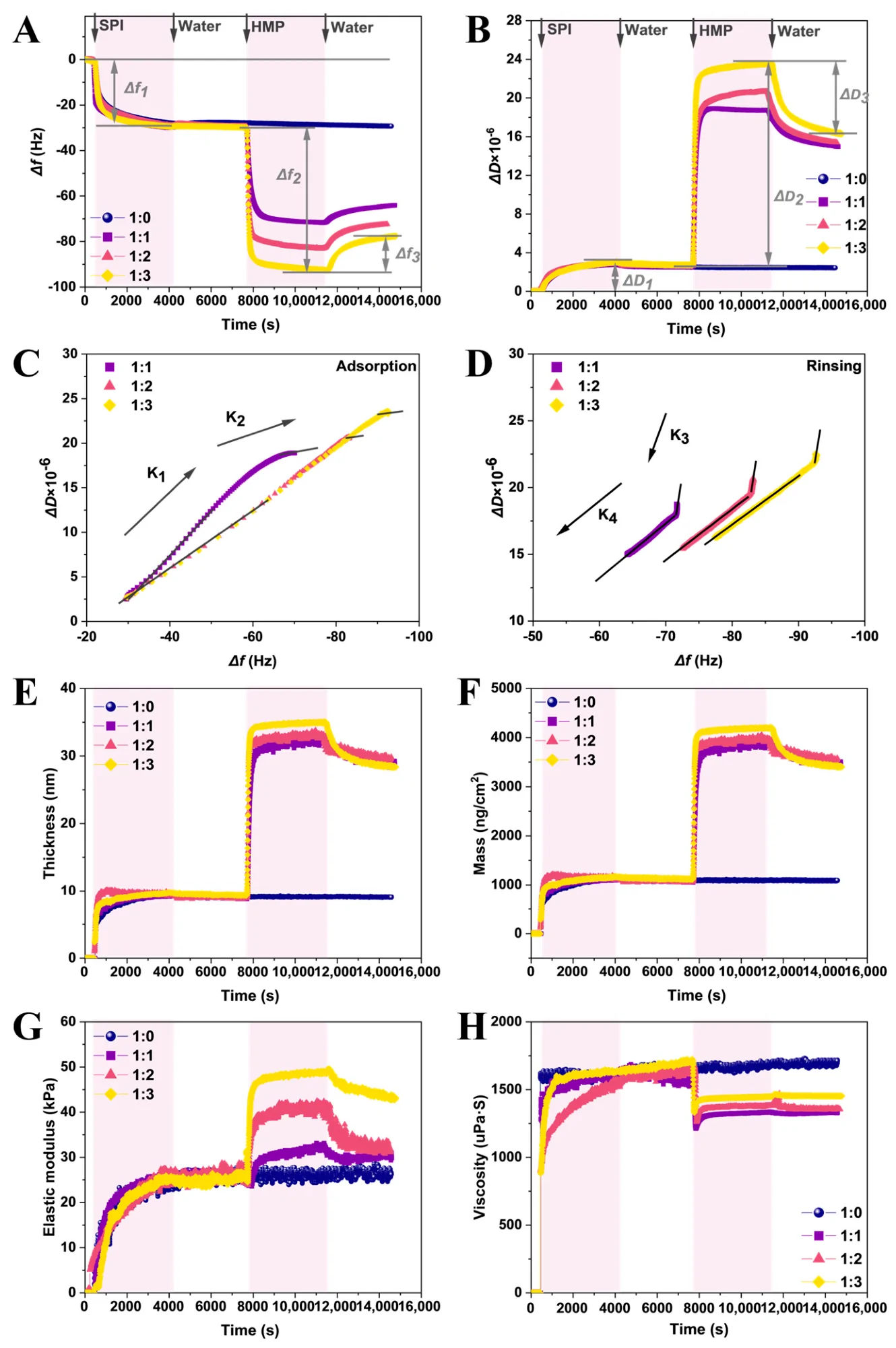
QCM-D Frequency change Δ*f* (**A**) and dissipation change Δ*D* (**B**), Δ*D*-Δ*f* curves (**C**) of adsorption and desorption at *n* = 3 overtones (**D**), thickness (**E**), mass (**F**), elastic modulus (**G**) and viscosity (**H**) of the interfacial layer of SPI/HMP solutions with varied ratios.

Initially, a stable baseline was established in the QCM-D instrument using pH 3.9 ultrapure water. Various emulsifiers were then introduced, with simultaneous monitoring of frequency shifts (Δ*f*) and dissipation shifts (Δ*D*) over time on the hydrophobic surface. The values of frequency and dissipation changes are summarized in Table 2. Figure 4 is QCM-D frequency variation Δ*f* (A) and dissipation variation Δ*D* (B), Δ*D*-Δ*f* curves of adsorption (C) and desorption (D) at *n* = 3, thickness (E), mass (F), elastic modulus (G), and viscosity (H) of SPI/HMP bilayer interfacial layer at different scales. During adsorption of all components, a decrease in Δ*f* accompanied by an increase in Δ*D* was observed (Figure 4A,B), indicating the deposition of hydrated emulsifier molecules and the formation of viscoelastic interfacial layers on the hydrophobic sensor surface. In the bilayer assembly process, the addition of SPI resulted in a rapid and significant decrease in Δ*f* accompanied by an increase in Δ*D*, confirming the stable attachment of SPI onto the hydrophobic surface. Subsequent the introduction of HMP caused further rapid and substantial decreases in Δ*f* and in-creases in Δ*D*, suggesting that HMP adsorbed onto the pre-adsorbed SPI layer and formed the secondary interfacial layer.

After the second rinsing, both Δ*f* and Δ*D* exhibited substantial reversal, indicating that the removal of loosely attached molecules and structural rearrangement of the adsorbed layer toward a more compact structure. Notably, both Δ*f*_2_ and Δ*f*_3_ showed greater magnitude changes with increasing HMP concentration. The maximum changes in Δ*f*_2_ and Δ*f*_3_ (63.164 Hz and 15.02 Hz, respectively) were observed at r = 1:3. This phenomenon may be attributed to strong electrostatic attraction between cationic SPI molecules and anionic HMP molecules, that significantly enhanced the interaction forces within the interfacial layer and interfacial coverage [44].

The high energy dissipation (Δ*D*) measured on the quartz crystal surface can be attributed to the formation of a soft, highly hydrated viscoelastic emulsifier layer [45]. As shown in Figure 4, all samples exhibited Δ*D* values exceeded 1 × 10^−6^, indicating the formation of viscoelastic interfacial layer [46]. Furthermore, the overtone-dependent QCM-D response further confirmed the characteristic behavior of a viscoelastic layer. Following SPI adsorption and subsequent HMP deposition, the Δ*D* values increased rapidly, demonstrating that incorporation of HMP significantly enhanced the hydration degree and structural heterogeneity of the interface. After adsorption was completed and the sensor was rinsed with deionized water, the Δ*D* values gradually decreased reached a stable plateau, suggesting the removal of loosely bound molecules and the formation of a denser interfacial structure through molecular rearrangement [47]. Also, Δ*D* increased with HMP concentration, reaching maximum values for Δ*D*_2_ and Δ*D*_3_ (20.5357 × 10^−6^ and 7.1941 × 10^−6^, respectively) at r = 1:3. This indicated that a higher HMP content promoted the formation of a thicker and more hydrated bilayer interface.

The Δ*D*-Δ*f* plot is widely used for qualitative analysis of conformational changes in adsorbed layer. A higher Δ*D*-Δ*f* value represents greater energy dissipation per frequency change, which is characteristic of softer, more hydrated interfacial structure [48]. As shown in Figure 4C,D, the discontinuous slope of the Δ*D*-Δ*f* curve suggested a conformational rearrangement during adsorption and desorption. Additionally, the widely distributed adsorption data points suggested rapid molecular attachment, whereas the relatively narrow desorption implied slower removal of adsorbed molecules [49]. From Table 3, it observed that for all SPI/HMP bilayer interfacial layers at different ratios, K_2_ is smaller than K_1_, suggesting that the initially flexible and highly hydrated adsorbed layer transited to a more compact organization.

The adsorption thickness and mass of the SPI/HMP bilayer interfacial layer (Figure 4E,F) were quantitatively evaluated using the Voigt model. Detailed quantitative data for adsorbed thickness and mass are summarized in Table 4. During adsorption, both interfacial thickness and adsorbed mass increased, indicating deposition of HMP molecules onto the pre-adsorbed SPI layer. As the HMP concentration increased, H_1_ and M_1_ values went upward. It was attributed to the fact that the negatively charged HMP tightly bind to SPI at the interfacial layer via electrostatic interactions, forming a stable and thick SPI/HMP bilayer and increasing interfacial coverage. During desorption, the adsorbed thickness and mass gradually decreased before stabilizing, indicating the removal of loosely bound molecules. The highest adsorption thickness (H_2_ = 29.59 nm) and adsorbed mass (M_2_ = 3543.8 ng/cm^2^) were observed at r = 1:2. Interestingly, the r = 1:3 sample exhibited a pronounced reduction in thickness and mass after rinsing, suggesting the additional material adsorbed at r = 1:3 was relatively loosely associated and readily removed, whereas r = 1:2 formed a more rinse-resistant and structurally persistent interfacial layer [50].

The changes of the elastic modulus and viscosity of the adsorption film with time during adsorption and desorption are shown in Figure 4G,H. From Table 5, during the adsorption process, both the elastic modulus and viscosity gradually increased, indicating that strengthening of the SPI/HMP interfacial structure. In contrast, the elastic modulus of the single-layer SPI is relatively low (E_2_ = 26.61 ± 0.24 kPa), implying that the relatively fragile protein layer. Following HMP adsorption, the interfacial mechanical strength improved due to the formation of the thicker interfacial layer and enhanced intermolecular interactions between HMP and SPI. During the desorption process, the decrease in viscosity is due to the denser adsorption of the multi-layer. The increase in elastic modulus reflected enhanced resistance to deformation, while the rise in viscosity indicated greater energy dissipation and increased structural complexity within the hydrated interface. The larger the elastic modulus and viscosity of the SPI/HMP double-layer interface layer, the stronger the binding force, facilitating the formation of an interfacial layer with higher viscoelasticity and more compacted structure.

Among all formulations, the emulsion r = 1:3 exhibited the highest viscoelastic parameters. It indicated that a higher HMP content reinforced the interfacial layer by enhancing intermolecular interactions and increasing surface coverage, improving the interface’s resistance to deformation and droplet destabilization. Although all emulsions remained viscous-dominated in bulk under the tested oscillatory conditions (tan δ > 1), HMP increased the retained viscoelastic properties of the adsorbed layer on the hydrophobic model surface. Together with the interfacial dilatational results, this enhanced interfacial response may contribute to resistance against droplet aggregation and coalescence [21].

## 4. Conclusions

The SPI/HMP bilayer emulsions with 5% (*v*/*v*) MCT as the oil phase were prepared via a two-step homogenization method, and the interfacial properties and emulsion stability at varied SPI/HMP mass ratios were investigated. Among them, r = 1:3, the emulsion exhibited the smallest droplet size (D _[3,2]_ = (3.974 μm), and Turbiscan Stability Index (TSI = 0.58), along with the highest apparent viscosity, storage modulus (G′) and loss modulus (G″) indicating its high stability. CLSM observations further confirmed the formation of a more homogeneous and compact droplet distribution.

QCM-D analysis revealed that at r = 1:3, the adsorbed interfacial layer demonstrated the strongest HMP deposition, strongest energy dissipation (Δ*D*_3_ = 7.1941 × 10^−6^), and greatest elastic modulus (43.15 kPa), with a viscosity of 1457.6 μPa·S. The results demonstrated the formation of a highly hydrated yet mechanically reinforced SPI/HMP interfacial layer. In contrast, at r = 1:2, the interfacial layer exhibited the highest adsorption thickness (H_2_ = 29.59 nm) and adsorbed mass (M_2_ = 3543.8 ng/cm^2^), indicating greater interfacial accumulation but not necessarily mechanical reinforcement.

Above all, the interfacial elastic modulus, adsorption affinity (Δ*f*_3_), and energy dissipation (Δ*D*_3_) showed a consistent increasing trend with improved emulsion stability. This implied that SPI/HMP bilayer emulsion stability was governed not only by the amount of interfacial deposition but also by the structural organization and mechanical strength of the interfacial layer. The enhanced elasticity and interfacial cohesion achieved at r = 1:3 contributed to improved resistance against droplet coalescence and long-term destabilization. Future studies should quantify non-adsorbed HMP in the separated serum phase, that would help distinguish continuous-phase kinetic arrest from interfacial stabilization.

## Figures and Tables

**Table 1:** D _[4,3]_, D _[3,2]_, Span and zeta potential of SPI/HMP bilayer emulsions at different ratios. Different letters indicated significant differences (*p* < 0.05).

SPI:HMP	D _[4,3]_ (μm)	D _[3,2]_ (μm)	Span	Zeta Potential
1:0	14.325 ± 0.107 ^d^	8.648 ± 0.056 ^d^	2.017 ± 0.020 ^d^	14.2 ± 0.4 ^d^
1:1	9.423 ± 0.041 ^c^	5.814 ± 0.030 ^c^	1.951 ± 0.018 ^c^	−19.6 ± 0.3 ^c^
1:2	8.158 ± 0.023 ^b^	4.323 ± 0.021 ^b^	1.861 ± 0.023 ^b^	−20.4 ± 0.4 ^b^
1:3	7.358 ± 0.026 ^a^	3.974 ± 0.027 ^a^	1.803 ± 0.019 ^a^	−21.1 ± 0.2 ^a^

**Table 2:** Frequency change Δ*f* and dissipation change Δ*D* during adsorption (Δ*f*_1_, Δ*D*_1_) and desorption (Δ*f*_2_, Δ*D*_2_) of SPI/HMP bilayer interfacial layer at different ratios. Values are expressed as mean ± standard deviation (*n* = 3 independent QCM-D measurements).

SPI:HMP	Δ*f*_1_ (Hz)	Δ*f*_2_ (Hz)	Δ*f*_3_ (Hz)	Δ*D*_1_ (×10^−6^)	Δ*D*_2_ (×10^−6^)	Δ*D*_3_ (×10^−6^)
1:0	27.726 ± 0.470	0.646 ± 0.164	0.665 ± 0.148	2.7987 ± 0.0764	0.3320 ± 0.0442	0.0227 ± 0.0072
1:1	29.936 ± 0.319	41.724 ± 0.087	7.661 ± 0.366	2.7875 ± 0.0576	15.9508 ± 0.0421	3.7700 ± 0.0293
1:2	28.926 ± 0.079	54.294 ± 0.603	10.87 ± 0.630	2.8688 ± 0.0253	17.7757 ± 0.0365	5.2655 ± 0.0144
1:3	29.416 ± 0.159	63.164 ± 0.415	15.02 ± 0.450	2.9888 ± 0.0346	20.5357 ± 0.0808	7.1941 ± 0.0179

**Table 3:** Slope of Δ*D*-Δ*f* curves during adsorption (K_1_, K_2_) and desorption (K_3_, K_4_) of SPI/HMP bilayer interfacial layer at different ratios. Values are expressed as mean ± standard deviation (*n* = 3 independent QCM-D measurements).

SPI:HMP	K_1_	K_2_	K_3_	K_4_
1:0	-	-	-	-
1:1	−0.4763 ± 0.0841	−0.0806 ± 0.0190	−2.1729 ± 0.3534	−0.3599 ± 0.0907
1:2	−0.3371 ± 0.0257	−0.1292 ± 0.0235	−3.0137 ± 0.3187	−0.3885 ± 0.0968
1:3	−0.3348 ± 0.0325	−0.1714 ± 0.0221	−1.6241 ± 0.0887	−0.3684 ± 0.0937

**Table 4:** Adsorbed thickness (H) and mass (M) during adsorption (H_1_, M_1_) and desorption (H_2_, M_2_) of SPI/HMP bilayer interfacial layer at different ratios. Values are expressed as mean ± standard deviation (*n* = 3 independent QCM-D measurements). Different lowercase letters within the same parameter and adsorption stage indicate significant differences among SPI ratios (one-way ANOVA followed by Tukey’s test, *p* < 0.05).

SPI:HMP	H_1_ (nm)	H_2_ (nm)	M_1_ (ng/cm^2^)	M_2_ (ng/cm^2^)
1:0	9.08 ± 0.21 ^a^	9.02 ± 0.12 ^a^	1095.3 ± 28.1 ^a^	1098.1 ± 28.6 ^a^
1:1	32.26 ± 0.22 ^b^	28.91 ± 0.22 ^b^	3858.4 ± 48.1 ^b^	3476.4 ± 38.5 ^b^
1:2	33.06 ± 0.24 ^c^	29.59 ± 0.32 ^c^	3963.3 ± 43.5 ^c^	3543.8 ± 52.1 ^c^
1:3	34.97 ± 0.27 ^d^	28.36 ± 0.26 ^b^	4191.7 ± 58.2 ^d^	3409.9 ± 61.2 ^b^

**Table 5:** The elastic modulus (E) and viscosity (V) during adsorption (E_1_, V_1_) and desorption (E_2_, V_2_) of SPI/HMP bilayer interfacial layer at different ratios. Values are expressed as mean ± standard deviation (*n* = 3 independent QCM-D measurements). Different lowercase letters within the same parameter and adsorption stage indicate significant differences among SPI ratios (one-way ANOVA followed by Tukey’s test, *p* < 0.05).

SPI:HMP	E_1_ (kPa)	E_2_ (kPa)	V_1_ (uPa·S)	V_2_ (uPa·S)
1:0	25.78 ± 0.23 ^a^	26.61 ± 0.24 ^a^	1688.5 ± 26.4 ^c^	1676.4 ± 14.5 ^c^
1:1	32.86 ± 0.51 ^b^	30.46 ± 0.71 ^b^	1331.9 ± 56.5 ^a^	1333.2 ± 51.3 ^a^
1:2	41.48 ± 0.61 ^c^	32.36 ± 0.52 ^c^	1381.3 ± 36.1 ^a^	1362.8 ± 51. 5 ^a^
1:3	49.13 ± 0.53 ^d^	43.15 ± 0.62 ^d^	1447.6 ± 53.2 ^b^	1457.6 ± 23.7 ^b^

## Data Availability

The original contributions presented in this study are included in the article. Further inquiries can be directed to the corresponding authors.
